# The self-assembly of l-histidine might be the cause of histidinemia

**DOI:** 10.1038/s41598-023-44749-5

**Published:** 2023-10-14

**Authors:** Ajitha Ajikumar, Anakha Kandara Nikarthil Premkumar, Sunilkumar Puthenpurackal Narayanan

**Affiliations:** NMR Facility, Institute for Integrated Programmes and Research in Basic Sciences, Mahatma Gandhi University, Priyadarshini Hills P. O., Kottayam, 686560 Kerala India

**Keywords:** Chemical biology, Neuroscience, Diseases

## Abstract

l-Histidine is an essential amino acid with unique biochemical and physiological properties. Histidinemia is a disease condition caused by the elevated level of l-histidine in our blood. Mutations in the histidase, an enzyme for the breakdown of histidine, is the cause of the rise in histidine concentration. To our knowledge, no research has been done on why a high concentration of histidine causes histidinemia. In this study, we provide a potential explanation why the elevated levels of histidine in the human body causes histidinemia. In this study we have found that l-histidine self-assembled in water to form nano sheet structures at physiological pH and temperature, using 1D ^1^H NMR spectroscopy, diffusion ordered spectroscopy (DOSY) and scanning electron microscope (SEM) techniques. The kinetics of self-assembly has been studied using real time NMR spectroscopy. We observed that both the aromatic ring and aliphatic part are equally contributing to the self-assembly of l-histidine. The symptoms of histidinemia, neurological deficits and speech delays, are similar to that of the neurodegenerative diseases caused by the self-assembly of peptides and proteins. We speculate that the self-assembly of l-histidine might be the cause of histidinemia.

## Introduction

l-Histidine (l-His, L-H) is an essential amino acid that is used in the production of proteins and frequently present at the active site of proteins^1^. It shows anti-inflammatory, anti-oxidant, and antisecretory functions within the body^2^. The genetic metabolic disorder known as histidinemia is defined by the increased amounts of the amino acid histidine in our body^3^. The primary signs of this disease are: (i) behavioural abnormality, which is an abnormality of mental functioning and includes a variety of affective, behavioural, cognitive, and perceptual abnormalities; (ii) hyperactivity, which is a state of being unusually or abnormally active, including in situations where it is inappropriate; (iii) moderate psychomotor retardation, which is a moderate delay in the achievement of motor or mental milestones in the development domains of a child; (iv) neurological speech impairment and (v) specialized learning disability, which are impairments of specific learning abilities like reading or writing, coordination, self-control, or attention^1^.

Urocanic acid is the product of breakdown of histidine by the enzyme histidase^4^. Mutation in the HAL gene, which gives instructions for producing the histidase enzyme, is the cause of the rise in histidine concentration and subsequently histidinemia^5^. Increased levels of histidine are seen in the blood and urine of histidinemia patients as a result of HAL gene mutations that produce an ineffective histidase enzyme. However, no research has been done on why a high concentration of histidine causes histidinemia, despite the fact that the molecular mechanism of the increase in histidine concentration has been well researched. In this study, we provide a potential explanation how unusually elevated levels of histidine in the human body causes the histidinemia illness condition. Neurological deficits include behavioural abnormalities, hyperactivity, psychomotor retardation, speech impairment, learning disability, etc. are present in people with histidinemia^1^. The symptoms of histidinemia resembles neurodegenerative amyloid disease, which develops when certain peptides and proteins self-assemble in our body and deposit as amyloid fibrils in the brain^6^.

According to extensive research histidine-containing peptides and proteins can self-assemble^7^. Prabhjot Singh et al. reported the self-assembly of l-histidine in water–methanol medium^8^. Using nuclear magnetic resonance (NMR) spectroscopy and scanning electron microscope (SEM) methods, we investigated the self-assembly of histidine in water, for the first time, at concentrations of 25 mM and 50 mM at physiological pH (7.5) and temperatures of 20 °C and 37 °C (physiological temperature). In this study we revealed that histidine performs self-assembly in an environment that is comparable to our physiological conditions: an aqueous medium at pH 7.5 and temperature 37 °C. Real-time NMR spectroscopy was used to investigate the kinetics of self-assembly. It has been noted that both the aliphatic carboxyl terminus and the aromatic imidazole ring contribute equally to the kinetics of histidine self-assembly. The development of self-assemblies with nano-sheet morphology was confirmed by SEM images. The diffusion ordered spectroscopy (DOSY) analysis result also supported the self-assembly of l-His in water. Blood histidine levels in histidinemia patients range from 290 to 1420 μΜ^3^. So, we have caried out DOSY experiment on 1 mM l-His in water, which is coming in the histidinemia concentration range, at pH 7.5 and temperature 37 °C from which the presence of more than one states in the system is very clear confirming the self-assembly of 1 mM l-His in water.We speculate that the self-assembly of histidine may be the source of histidinemia, a condition brought on by a larger concentration of histidine in our body that shares symptoms with neurodegenerative amyloid disorders. To the best of our knowledge, no other study of the self-assembly of l-histidine in water, its kinetics and also the reason why a higher concentration of histidine leads to histidinemia have been reported yet.

## Results

### NMR spectroscopy confirmed the self-assembly of l-His in water

**Figure 1:**
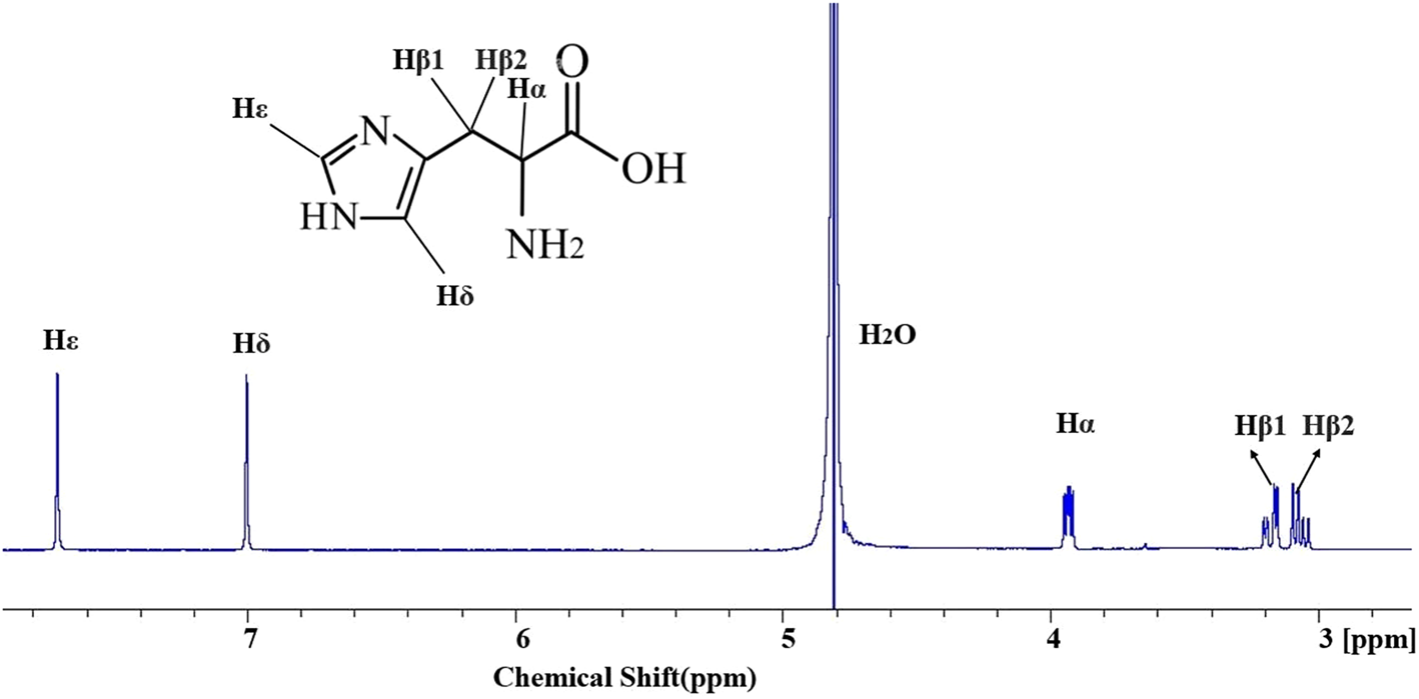
The assigned 1D ^1^H NMR spectrum of 25 mM l-His in water at pH 7.5 and temperature 20 °C.

**Figure 2:**
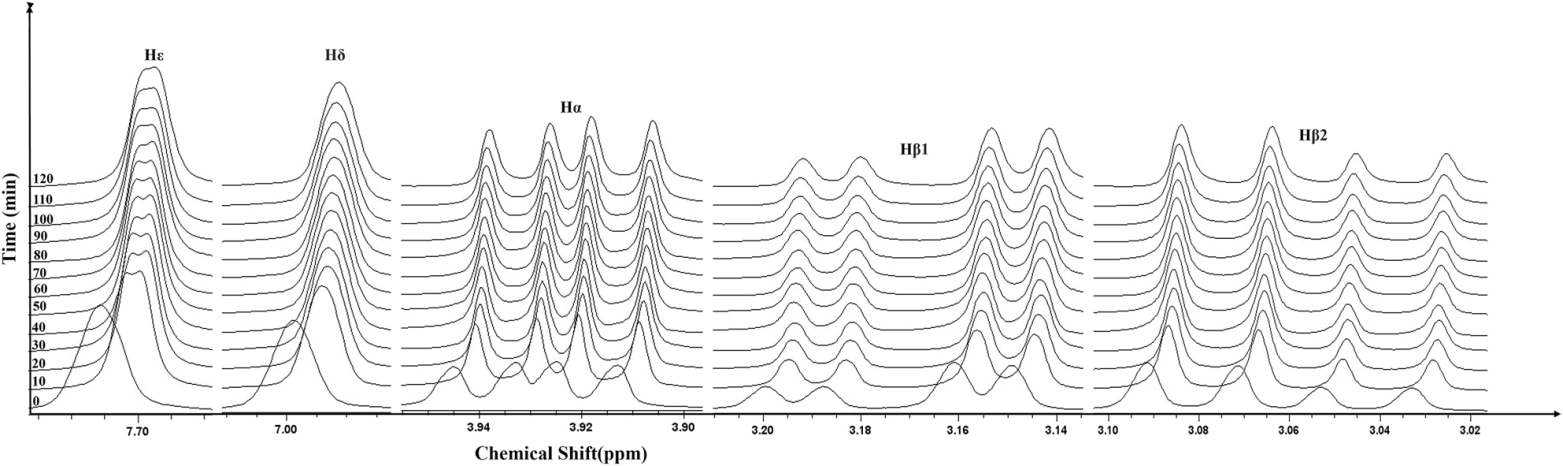
An overlay of time dependent 1D ^1^H NMR spectra of 25 mM l-His in water at pH 7.5 and temperature 20 °C.

A very promising tool for identifying and characterising molecule self-assembly is NMR spectroscopy. The molecules in the self-assembled state have a different NMR peak position (chemical shift) than those in the free state. Figure 1 depicts the assigned 1D ^1^H NMR spectrum of 25 mM l-His in water at pH 7.5 and 20 °C. First, we acquired the time-dependent 1D ^1^H NMR spectra of 25 mM l-His in water at pH 7.5 and temperature 20 °C. Figure 2 displays an overlay of time-dependent 1D ^1^H NMR spectra. We saw that the peak positions (chemical shifts) moved up field with time and stabilised at about 120 min (7200 s). The sample solution was clear indicating that no precipitation has occurred. Only the self-assembly of His molecules into a well-ordered structure, in which the protons are more shielded than the free molecules, could account for the potential cause of the chemical shift change. We obtained FE SEM images of the sample in order to understand the state of the amino acid molecules inside the solution, and we discovered that l-His self-assembled into well-ordered nanostructures (to be discussed in more detail later).

### Kinetics of self-assembly of l-His in water from real time NMR

**Figure 3:**
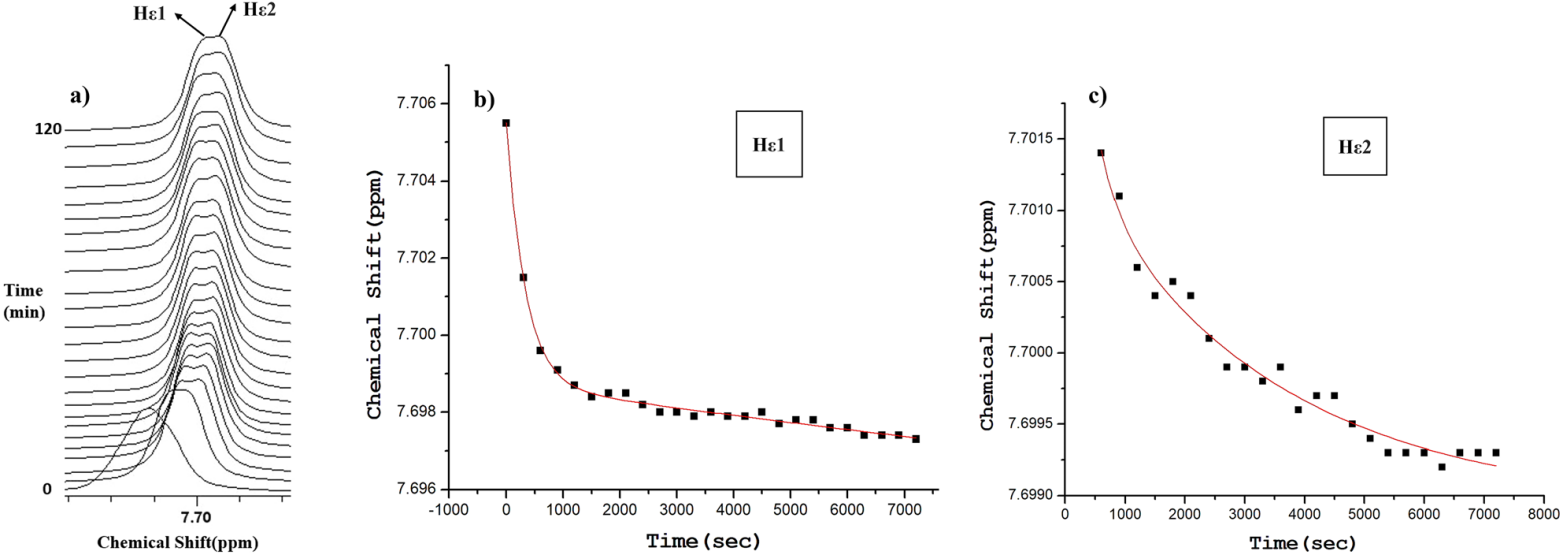
(**a**) An overlay of the Hɛ proton peaks of the 25 mM l-His in water at pH 7.5 and temperature 20 °C as a function of time. (**b**) A plot of the chemical shift values of the Hɛ1 peak as a function of time. (**c**) A plot of the chemical shift values of the Hɛ2 peak as a function of time. Biexponential decay kinetic equation [Eq. (1) in the methods section] has been fitted to the chemical shift plots.

**Figure 4:**
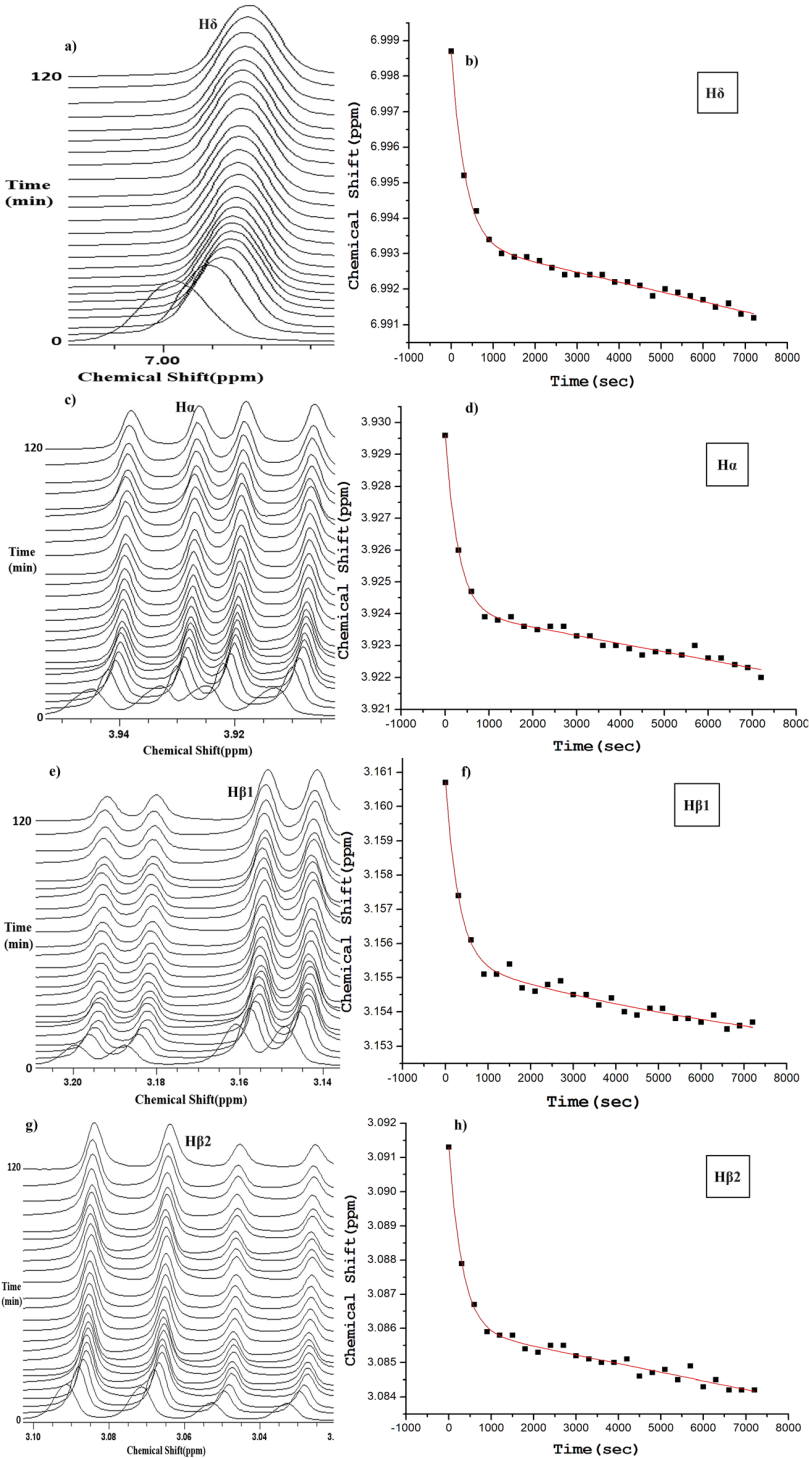
An overlay of time dependent NMR peaks of different protons, other than Hɛ, of 25 mM l-His in water at pH 7.5 and temperature 20 °C, and the corresponding chemical shift plots as a function of time are shown in this figure. (**a**, **b**) Hδ proton, (**c**, **d**) Hα proton, (**e**, **f**) Hβ1 proton, and (**g**, **h**) Hβ2 proton. Biexponential decay kinetic equation [Eq. (1) in the methods section] has been fitted to all the chemical shift plots.

Figure 3a shows an overlay of the Hɛ proton peaks of the 25 mM l-His in water at pH 7.5 and temperature 20 °C and Fig. 3b displays the chemical shift values as a function of time. It is evident that a biexponential decay pathway, with a quick initial phase and a slow second phase, was followed by the change in chemical shift values. We fitted the biexponential decay equation [Eq. (1) in the materials and methods] to the plot of the variations in chemical shift as a function of time (Fig. 3b) in order to derive the rate constants for the self-assembly. It is fascinating to note that the Hɛ peak began to split around 10 min/600 s, indicating the existence of a second pathway for self-assembly in which the Hɛ proton has a different chemical environment (Fig. 3a–c). With the peaks from the remaining protons, Hδ, Hβ1, Hβ2 and Hα, we repeated the process (Fig. 4a–h). Table 1 lists the rate constants that were found from various l-His protons. The fact that rate constants from all of the protons, with the exception of the second Hɛ peak, are comparable is another intriguing finding. Self-assembly formation is responsible for peak broadening and decrease in signal intensity. The spectra clearly show that peak broadening and intensity reduction are happening as a function of time. Since there is no general simple relationship between the self-assembly and the degree of line broadening or intensity reduction, we have not used these changes to estimate the rate of self-assembly (please see the explanation given in the Supplementary Materials).

**Table 1:** Rate constants of self-assembly of l-His in water at all the experimental conditions.

Atom	Rate constant (s^−1^)	
	k1	k2
(a)		
Hɛ1	0.0030	0.000026
Hɛ2	0.0030	0.0003
Hδ	0.0030	0.000026
Hβ1	0.0030	0.000026
Hβ2	0.0030	0.000026
Hα	0.0030	0.000026
(b)		
Hɛ		0.00043
Hδ		0.00043
Hβ1		0.00043
Hβ2		0.00043
Hα		0.00043
(c)		
Hɛ		0.00056
Hδ		0.00054
Hβ1		0.00056
Hβ2		0.00056
Hα		0.00055
(d)		
Hɛ		0.00068
Hδ		0.00067
Hβ1		0.00068
Hβ2		0.00068
Hα		0.00068

(a) 25 mM at pH 7.5 and 20 °C. (b) 50 mM at pH 7.5 and 20 °C. (c) 25 mM at pH 7.5 and 37 °C. (d) 50 mM at pH 7.5 and 37 °C.

### Effect of concentration on the rate of self-assembly of l-His in water

We conducted real-time 1D ^1^H NMR experiments with 50 mM l-His in water at pH 7.5 and temperature 20 °C in order to comprehend the impact of concentration on the rate of l-His self-assembly. In Fig. S1 of the Supplementary Materials, an overlay of time-dependent 1D ^1^H NMR spectra is displayed. We saw that the peak positions (chemical shifts) moved up field with time and stabilised at about 120 min. It is obvious that a monoexponential decay pathway was followed by the change in chemical shift values. We fitted the monoexponential decay equation [Eq. (2) in the Experimental Section] to the plot of the changes in chemical shift in order to derive the rate constants for self-assembly (Fig. S2a–j). The rate constants that were found from various l-His protons are listed in Table 1. It is interesting to note that the rate constants from all the protons are comparable.

### Effect of temperature on the rate of self-assembly of l-His in water

We conducted real-time NMR studies at 37 °C (physiological temperature) with 25 mM and 50 mM l-His in water with a pH of 7.5 to better understand how temperature affects the rate of l-His self-assembly. Figures S3a and b in the Supplemental Materials display the overlays of time-dependent 1D ^1^H NMR spectra at 25 mM and 50 mM concentrations. We saw that the peak positions (chemical shifts) moved up field with time and stabilised at about 120 min. It is evident that a monoexponential decay pathway was followed by the change in chemical shift values. We fitted the mono exponential decay equation [Eq. (2) in the Experimental Section] to the plot of the changes in chemical shift as a function of time (Figs. S4a–j and S5a–j) in order to derive the rate constants for the self-assembly. Table 1 lists the rate constants that were found from various l-His protons. It is interesting to note that the constants from all the protons under the same circumstances are comparable.

### FE SEM analysis of the self-assembly of l-His in water

**Figure 5:**
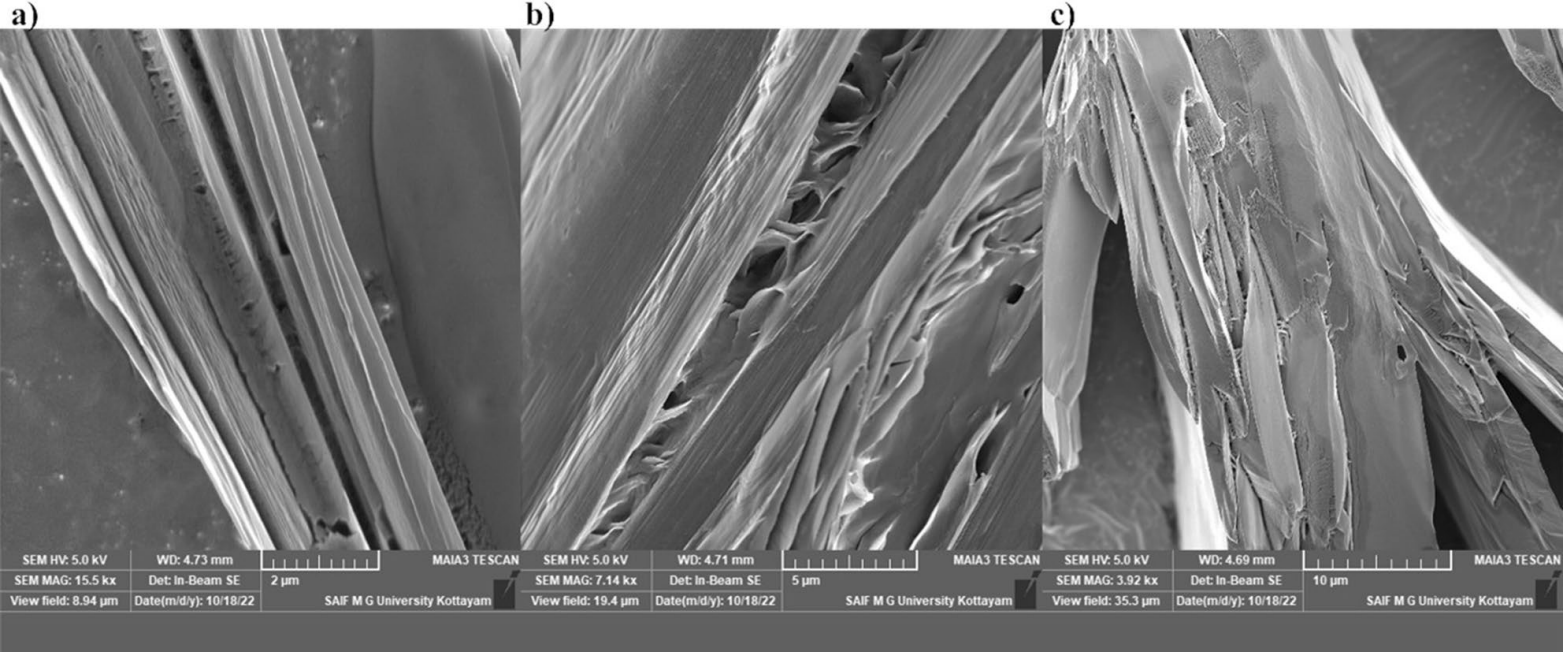
The SEM images of 25 mM l-His in water at pH 7.5 and 20 °C at different resolutions (**a**) 2 µm (**b**) 5 µm (**c**) 10 µm.

In the NMR spectroscopy study, the chemical shift changes as a function of time became insignificant after 2 h. The scanning electron microscopy (SEM) images of 25 mM l-His sample in water at a pH of 7.5 and a temperature of 20 °C were recorded for verifying the self-assembly process and for understanding the morphology of the self-assemblies. The SEM images with different resolutions are shown in Fig. 5. It is evident that histidine self-assembled into sheet-like nanostructures in water, having similar morphology as that created in the water–methanol solvent^8^.

### Diffusion ordered spectroscopy (DOSY) study of l-His in water

Diffusion ordered spectroscopy (DOSY) is a well-established NMR method to study the self-assembly of biomolecules^9^. Different states present in the solution, monomers and self-assemblies, can be identified based on their diffusion coefficients. We have carried out DOSY experiment on 25 mM l-His in water at pH 7.5 and temperature 37 °C after two hours of incubation. The pseudo-2D DOSY spectrum of the sample is shown in Fig. S6 in the Supplementary Material, from which the presence of more than one states in the system is very clear further confirming the self-assembly of L-His in water. Blood histidine levels in histidinemia patients range from 290 to 1420 μΜ (normal 70–120 μΜ). So, we have caried out DOSY experiment on 1 mM l-His in water, which is coming in the histidinemia concentration range, at pH 7.5 and temperature 37 °C after 24 h of incubation. The pseudo-2D DOSY spectrum of the 1 mM l-His sample is shown in Fig. S7 in the Supplementary Material, from which the presence of more than one states in the system is very clear confirming the self-assembly of 1 mM l-His in water, which is coming in the histidinemia concentration range.

## Discussion

Molecules spontaneously arrange themselves into bigger structures or patterns through a process known as molecular self-assembly, without the aid of outside guidance or interference^10^. The intermolecular interactions between molecules, which can be electrostatic, hydrogen bonds, van der Waals forces, or covalent bonds, are what cause this behaviour^11^. Self-assembly is a fundamental process in nature that creates a variety of complex structures, including DNA helices, protein complexes, and lipid bilayers in cell membranes^12^. As a potent tool for the development of novel materials with specialised features, such as nanoscale electrical devices, drug delivery systems, and sensors, molecular self-assembly is also of interest to scientists^11^.

Deficiency as well as excess of amino acids in our blood cause disease conditions. Amino acids are the smallest biomolecules showing self-assembly. It has been reported that out of twenty naturally occurring amino acids ten amino acids show self-assembly which are phenyl alanine^13^, tryptophan^14^, histidine^14^, glycine^14^, alanine^14^, valine^14^, serine^14^, isoleucine^14^, proline^14^ and lysine^14^. Higher levels of amino acids in our blood cause a disease condition which can be generally termed as “hyperaminoacidemia” with a few exceptions like phenylketonuria (high level of phenyl alanine) and histidinemia (high level of histidine)^3,15,16^. All these diseases as a result of high amino acid levels in our blood have the symptoms of neurological disorders like developmental delays, intellectual incapacity, seizures, mobility abnormalities etc.^5,14,15^. It has been well studied that the mutations on the enzymes catalysing the breakdown of amino acids are responsible for the high levels of amino acids in our body^5,15^. It has been reported that high concentration of phenyl alanine in our body leads to a neurodegenerative disease called phenylketonuria, which is a consequence of self-assembly of phenyl alanine^15^. The abnormal build-up of Tryptophan is known to cause hypertryptophanimea, a neurodegenerative disease caused by the self-assembly of tryptophan^17^. In the case of all other amino acids, the molecular mechanism of diseases caused by their excess levels in our body is not well studied.

L-histidine is an amino acid that is necessary for human nutrition and has a number of medically proven advantages^2^. Increased dietary histidine intake and supplementation at doses of 4.0–4.5 g histidine/day are linked to lower levels of oxidative stress, proinflammatory cytokines, fasting blood sugar, and markers of glucose homeostasis^18^. Intake of histidine also enhances cognitive performance (e.g., decreases appetite, anxiety and stress, and improves sleep); potentially through the metabolism of histidine to histamine, nevertheless, the relationship between histidine and histamine in people is unclear^18^. Studies have found negative effects of histidine at high intakes (> 24 g/day), including lower serum zinc levels and cognitive impairment^18^.

High amounts of histidine in our blood lead to the development of a rare hereditary disorder known as histidinemia^3^. People with histidinemia are unable to adequately break down histidine, thus it builds up in their blood and urine^3,19^. The body needs histidine, an essential amino acid, to build and repair its tissues on a daily basis. Histidine and its metabolites, however, can be harmful to the body in large doses and result in symptoms like behavioural issues, speech and language problems, intellectual disability, and developmental delays^3,20^. Histidinemia is caused by mutations in the gene that generates the enzyme histidase, which breaks down histidine, which causes histidine to accumulate^3,21^. Histidinemia is said to be caused by an excess of histidine in the blood, however it has not yet been shown why a high quantity causes histidinemia. In this study, utilising 1D ^1^H NMR spectroscopy, DOSY and SEM as analytical techniques, we report for the first time the self-assembly of the amino acid l-histidine in aqueous medium at physiological pH and temperature. All of these analytical methods demonstrated that L-histidine self-assembles in a condition resembling a physiological one.

The chemical shift value of a nucleus in NMR spectroscopy is dependent on its environment, hence a change in chemical shift denotes a change in environment. In the process of self-assembly, molecules are organised into certain morphologies, such as fibrils, sheets, tubes, and rods, among others^14^. Non-covalent interactions, such as hydrogen bonds, van der Waals contacts, pi-pi stacking, electrostatic interactions, and hydrophobic interactions, stabilise the self-assemblies^11^. The surroundings of the monomer molecule nuclei are altered by each of these interactions. Real-time 1D ^1^H NMR spectroscopy has been employed in this study to better understand the self-assembly process. The self-assembly process is readily visible in our observations of the peak positions or chemical shifts of the protons in the His molecule as a function of time. It is evident that under the same experimental conditions, all of the protons followed exponential decay trajectories at rates that were comparable. By fitting exponential kinetic equations to the chemical shift changes as a function of time as stated in the methodology and results sections, it was possible to determine the rate of L-His self-assembly in water. The Hɛ proton peak split at a concentration of 25 mM l-His at 20 °C, and the second peak has a different rate constant. Based on the available information, the possible explanation for the Hɛ peak splitting as a function of time is that two alternative pathways of self-assembly occur for which the Hɛ proton has different environments. The most crucial finding is that the self-assembly process is equally influenced by non-covalent interactions such as hydrophobic interactions, hydrogen bonds, electrostatic interactions, and pi-pi stacking. Both increasing concentration and temperature sped up the rates of self-assembly (see Table 1). An increase in rate of self-assembly by an increase in concentration is because of the increased number of l-His molecules available for the self-assembly. An increase is rate of self-assembly of l-His by an increase in temperature from 20 to 37 °C is because of the increase in hydrophobicity of the amino acid by temperature^22^, hydrophobic interactions have crucial role in self-assembly. For each proton, a biexponential route with a fast initial phase and a slow second phase was found at 25 mM l-His concentration and 20 °C temperature. Since only the slow phase could be detected by NMR at higher temperatures and concentrations, comparisons are based on the rates of the slow phase that could be detected. DOSY experiments proved the occurrence of more than one states, monomer and self-assemblies, in aqueous solutions of l-His.

The scanning electron microscopy (SEM) is a very helpful tool for verifying the self-assembly process and for understanding the morphology of the self-assemblies. After two hours, the time-dependent chemical shift changes became minor, and we obtained the SEM images of the 25 mM l-His sample in water with a pH of 7.5 and a temperature of 20 °C. It is obvious that histidine self-assembled into sheet-like nanostructures in water, mimicking the shape of structures created in a water–methanol solvent^8^. All of the experimental methods utilised in this study, as was already discussed, demonstrated that l-histidine self-assembled in aqueous medium. This is a convincing justification for why a high level of l-histidine in human blood creates histidinemia, a condition with symptoms similar to those of other neurodegenerative illnesses. We carried out time dependent 1D ^1^H NMR and SEM experiments on l-Phe, l-Trp and Gly in water at similar conditions as that of l-His. We observed that these amino acids are also undergoing self-assembly in water, but with different rates. l-Phe formed nano-fibrils and others formed nano-sheet structures (unpublished results). It has already been reported that self-assembly of l-Phe is responsible for the neurodegenerative disease called phenylketonuria^15^ and self-assembly of l-Trp is responsible for the neurodegenerative disease called hypertryptophanimea^17^.

Proteins, peptides, and amino acids self-assemble in several neurological diseases like Alzheimer's, Parkinson's, Type II diabetes, and phenylketonuria^23^. Alzheimer's disease, Parkinson's disease, age-related macular degeneration (AMD), and cataracts are all diseases caused by protein self-assembly^24^. The self-assembly of Aβ peptides causes Alzheimer's disease^25^. Excess of amino acids in our blood leads to disease conditions having symptoms similar to that of neurodegenerative diseases. It has been well demonstrated that the diseases like phenylketonuria (excess of phenyl alanine), tyrosinemia (excess of tyrosine), hypertryptophanimea (excess of tryptophan) are arising as a result of amino acid self-assembly. For the first time we report that l-histidine can self-assemble at conditions similar to physiological condition. Blood histidine levels in histidinemia patients range from 290 to 1420 μΜ^3^. So, we have caried out DOSY experiment on 1 mM l-His in water, which is coming in the histidinemia concentration range, at pH 7.5 and temperature 37 °C from which the presence of more than one states in the system is very clear confirming the self-assembly of 1 mM l-His in water. We speculate that the disease histidinemia, arising as a result of high level of l-histidine in our blood and having symptoms of neurological disorders, is the result of self-assembly of the l-histidine.

## Methods and protocols

### Materials

The amino acid l-Histidine (with 99% purity) was purchased from NICE Chemicals Pvt. Ltd. And was directly used for preparing sample solutions in deionized Millipore water. The pH of aqueous solutions of His was measured using a pH paper (purchased from NICE chemicals Pvt. Ltd.). The deuterium oxide required for preparing NMR samples was purchased from ALDRICH. The NMR spectra were recorded using 400 MHz Bruker Avance III spectrometer. High precision 5 mm NMR sample tubes were used for the analysis. The NMR data were processed and analysed using Topspin 4.1.4 software, developed by Bruker BioSpin GmbH (https://www.bruker.com>nmr-software>topspin). The time dependent chemical shift data were plotted and analysed using the Origin 6.0 software^26^. Morphology images of the self-assemblies was obtained by using the Tescan MAIA3 XMH Field Emission Scanning Electron Microscope (FE SEM) instrument^27^. The DOSY spectrum was analysed using the Dynamics Center software from Bruker.

### Methods

We have identified and characterised the self-assemblies of l-His in water under different conditions using NMR spectroscopy and FE SEM techniques. The kinetics of self-assembly was obtained from real time NMR spectra.

#### ***1D ***^***1***^***H NMR study***

The NMR analyses of l-His samples were done under four different conditions: (i) 25 mM l-His in water at pH 7.5 and temperature 20 °C, (ii) 25 mM l-His in water at pH 7.5 and temperature 37 °C, (iii) 50 mM l-His in water at pH 7.5 and temperature 20 °C and (iv) 50 mM l-His in water at pH 7.5 and temperature 37 °C. 600 µL of each sample containing 5% D_2_O, for locking purpose, was used for recording 1D ^1^H NMR spectra. NMR spectroscopy is an efficient tool for understanding the self-assembly of molecules. The NMR parameter used for understanding the self-assembly is the chemical shift (δ) value, which indicates the chemical environment of an NMR active nucleus. In this study, we have recorded 100 numbers of 1D ^1^H spectra of the l-His solution as a function of time, the experimental time of each experiment was 5 min (300 s) and hence the total experimental time was 8 h 20 min. Initially, 1D ^1^H NMR spectra were recorded using the simple pulse programme ‘zg’ (Bruker pulse program database). The solvent used was 95% H_2_O and 5%D_2_O, so the spectrum gave only a large water peak. For obtaining peaks from the sample (His) we used another pulse program, ‘zgpr’, with water suppression, which gave sample peaks with good signal to noise ratio (Bruker pulse program database). The acquisition parameters used were, spectral width (SW) 15 ppm, and number of data points (TD) 64 k. The spectra were processed by applying zero filling (64 k) and the window function exponential multiplication (em). All the spectra were referenced with respect to the water peak (4.8 ppm).

#### Analysis of time dependent 1D ^1^H NMR data

The chemical shifts of both aromatic and aliphatic proton peaks were picked from each spectrum using the Topspin program. The chemical shift values were plotted as a function of time using the Origin 6.0 program. The rate constants for the self-assembly of histidine was obtained by fitting the rate equation to the changes in chemical shifts as a function of time. The chemical shift changes followed biexponential and monoexponential decay pathways.

The biexponential decay function [Eq. (1)] used to fit on the chemical shift data is the following

1 $$\delta \, = \, \delta {1 } + {\text{A1 exp }}\left( { - {\text{t}}/{\text{t1}}} \right) \, + {\text{ A2 exp }}\left( { - {\text{t}}/{\text{t2}}} \right)$$

where, δ is the chemical shift at time t, δ1 is the chemical shift at the starting time, A1 is the net change in chemical for the first phase, A2 is the net change in chemical for the second phase, t is the time, t1 is the decay time constant for the first phase, t2 is the decay time constant for the second phase.

The rate constant for the first phase, k1 = 1/t1.

The rate constant for the second phase, k2 = 1/t2.

The monoexponential decay function [Eq. (2)] used to fit on the chemical shift data is the following

2 $$\delta \, = \, \delta {1 } + {\text{A1 exp }}\left( { - {\text{t}}/{\text{t1}}} \right)$$

where, δ is the chemical shift at time t, δ1 is the chemical shift at the starting time, A1 is the net change in chemical shift during the entire process, t is the time, t1 is the decay time constant.

The rate constant k = 1/t1.

#### FE SEM study

Significant chemical shift changes were observed during the first 2 h time. SEM images of the 25 mM l-His at pH 7–8 and temperature 20 °C were obtained after 2 h time. Sample was mounted with double sided carbon tape on aluminium stubs. The specimen was sputtered with thin layer of gold in auto fine coater and the images were examined at an accelerating voltage of 15 kV. Images with 2 µm, 5 µm and 10 µm resolutions were plotted.

### Diffusion ordered spectroscopy (DOSY) study

Diffusion ordered spectroscopy (DOSY) experiment was carried out on 25 mM and 1 mM l-His in water at pH 7.5 and temperature 37 °C using 400 MHz Bruker Avance III NMR spectrometer. The pulse sequence used was stebpesg1s, with excitation sculpting for water suppression. The field gradient was increased from 5 to 95% in 16 steps. The diffusion delay (Δ or d20) used was 0.1 s and the diffusion gradient length (δ or p30) used was 600 microseconds. The DOSY spectra were analysed using the Dynamics Center software from Bruker. The number of scans (ns) used for the 25 mM sample is 32 and for the1mM sample is 1024.

### Supplementary Information

Supplementary Figures.

Supplementary Information 2.

Supplementary Information 3.

## Data Availability

All data generated or analysed during this study are included in this published article [and its Supplementary Information files].
