# Supplementary material for: The self-assembly of l-histidine might be the cause of histidinemia
Source: Sci Rep. 2023 Oct 14;13:17461. doi: 10.1038/s41598-023-44749-5 (PMC10576791; doi:10.1038/s41598-023-44749-5)
Supplement: Supplementary file 1 — Supplementary Figures. [file 41598_2023_44749_MOESM1_ESM.docx]

**Supplementary Materials**

**The self-assembly of L-histidine might be the cause of histidinemia**

Ajitha Ajikumar^ǂ^, Anakha Kandara Nikarthil Premkumar^ǂ^ and Sunilkumar Puthenpurackal Narayanan*

NMR Facility, Institute for Integrated Programmes and Research in Basic Sciences, Mahatma Gandhi University, Priyadarshini Hills P. O., Kottayam-686560, Kerala, India.

^ǂ^ Equal contributions
*Correspondence: Dr. Sunilkumar Puthenpurackal Narayanan
 E-mail: [sunilkumarpn@mgu.ac.in](mailto:sunilkumarpn@mgu.ac.in)

**Comments on peak broadening and intensity reduction in NMR spectra**

Peak broadening and signal intensity reduction can be attributed to the formation of self-assembly. The linewidth of an NMR signal depends on the spin-spin relaxation time, T2, which, in turn, depends on the rotational correlation time, effectively the tumbling rate, of the molecule or molecular segment giving rise to an NMR signal. Generally, slower tumbling gives shorter T2, which in turn corresponds to broader signals. However, depending on the character of the self-assembled structure formed, the T2 of a signal may be controlled largely by local mobility rather than by the tumbling rate of the aggregate as a whole. Thus, there is no general, simple relationship between self-assembly size and the extent of line broadening or intensity reduction (1).

**
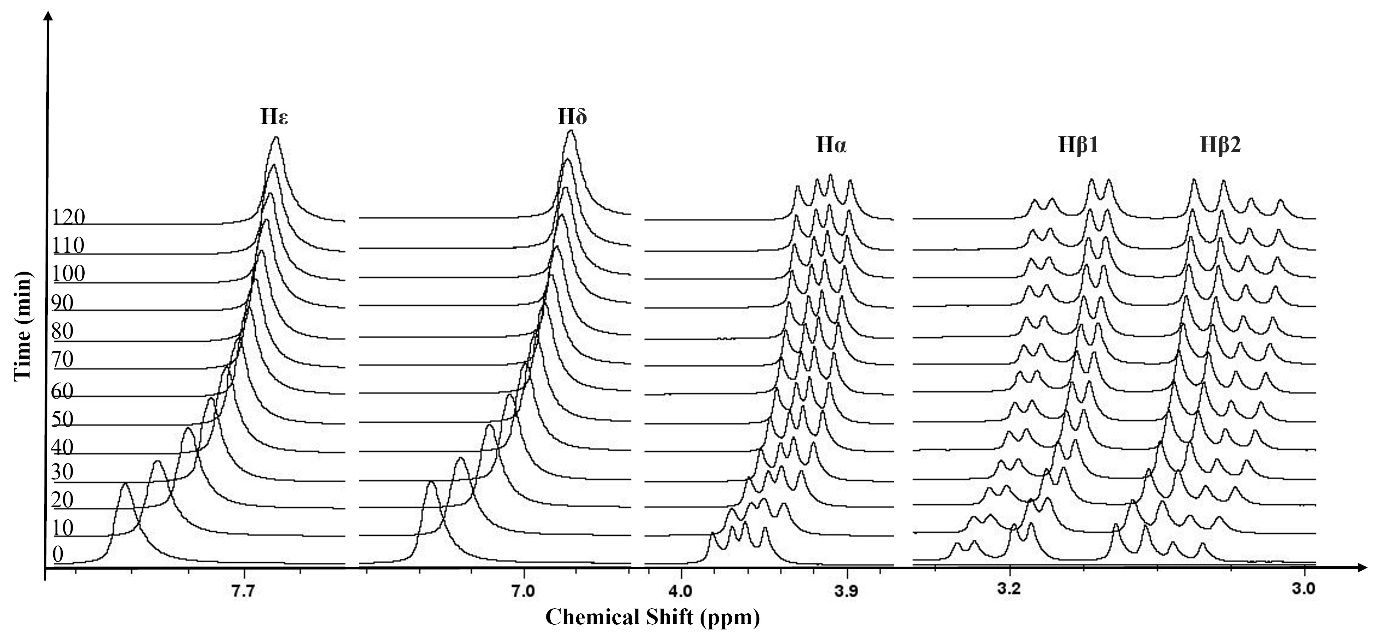
**

**Figure S1:** An overlay of time dependent 1D ^1^H NMR spectra of 50mM L-His in water at pH 7.5 and temperature 20ºC.

**
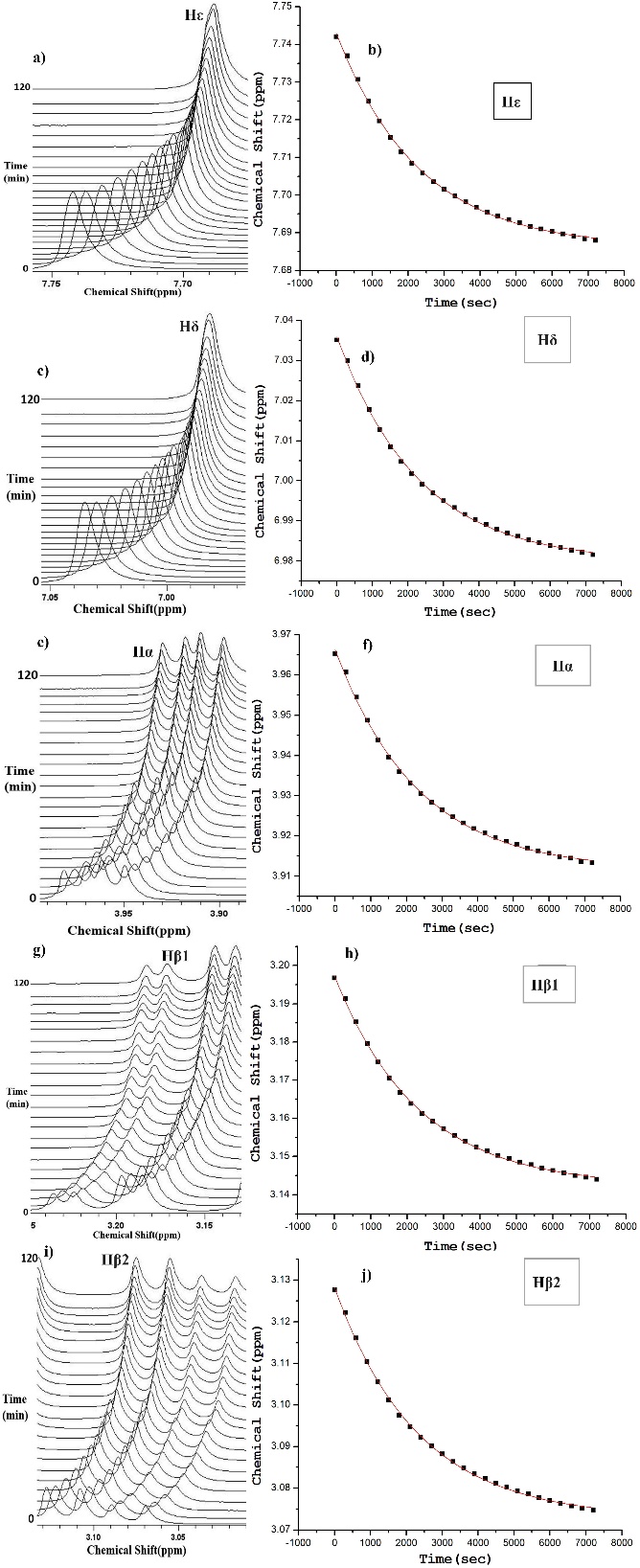
**

**Figure S2:** An overlay of time dependent NMR peaks of different protons of 50mM L-His in water at pH 7.5 and temperature 20ºC, and the corresponding chemical shift plots as a function of time are shown in this figure. (**a** & **b)** Hɛ proton, (**c** & **d**) Hδ proton, (**e & f**) Hβ1 proton, (**g & h**) Hβ2 proton and (**i** & **j**) Hα proton. Monoexponential decay kinetic equation (equation 2 in the methods section) has been fitted to all the chemical shift plots.

**
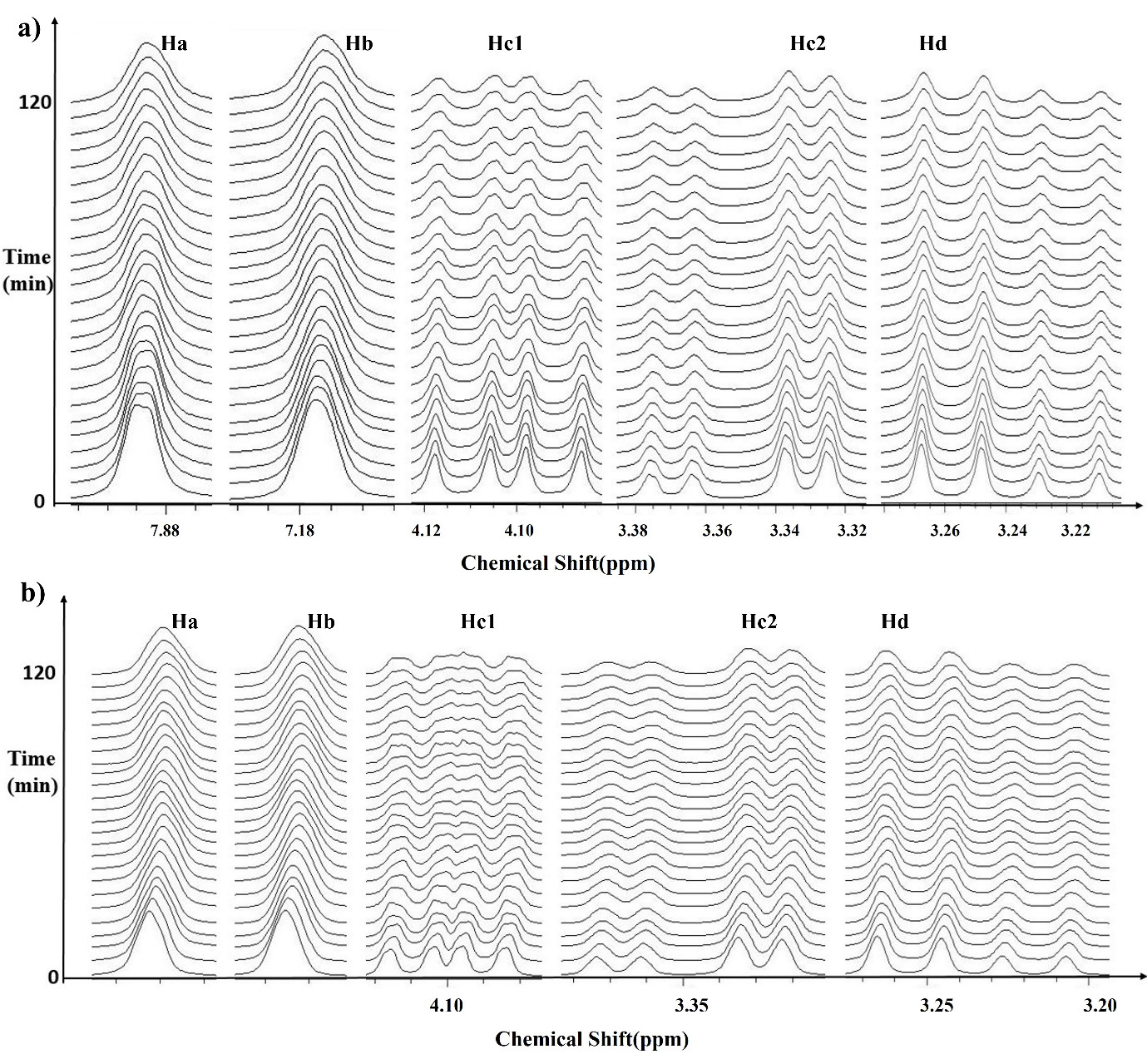
**

**Figure S3**: An overlay of time dependent 1D ^1^H NMR spectra at temperature 37ºC and pH 7.5 of (a) 25 mM L-His in water and (b) 50 mM L-His in water.

**
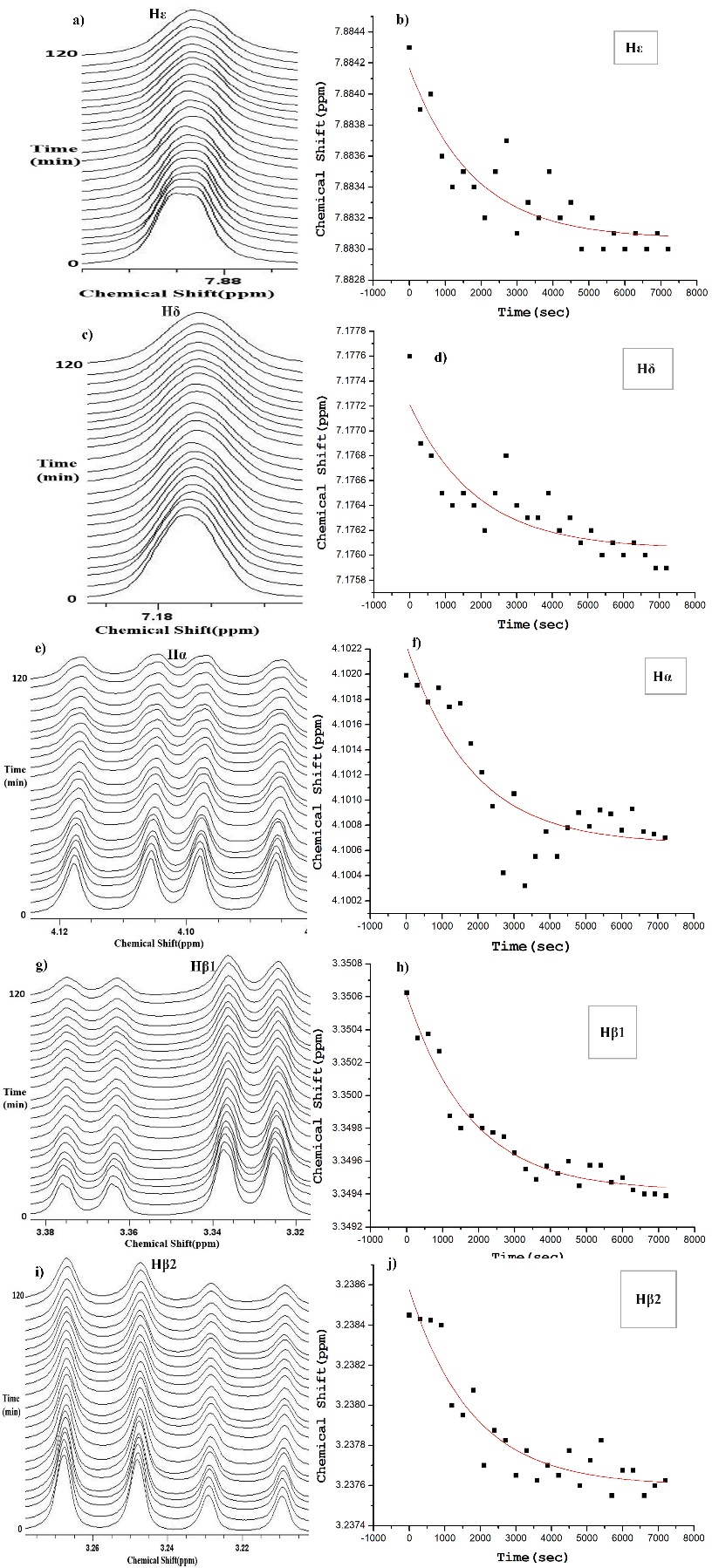
**

**Figure S4:** An overlay of time dependent NMR peaks of different protons of 25mM L-His in water at pH 7.5 and temperature 37ºC, and the corresponding chemical shift plots as a function of time are shown in this figure. (**a** & **b)** Hɛ proton, (**c** & **d**) Hδ proton, (**e & f**) Hβ1 proton, (**g & h**) Hβ2 proton and (**i** & **j**) Hα proton. Monoexponential decay kinetic equation (equation 2 in the methods section) has been fitted to all the chemical shift plots.

**
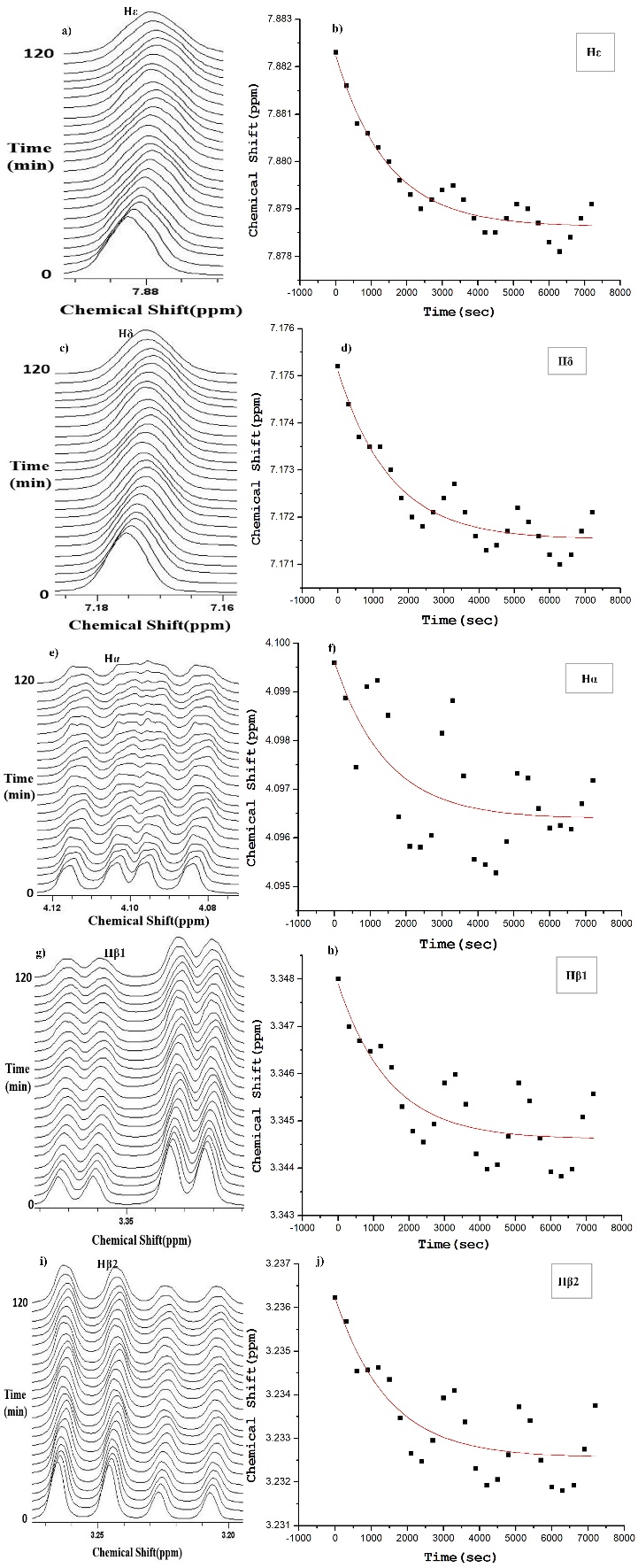
**

**Figure S5:** An overlay of time dependent NMR peaks of different protons of 50mM L-His in water at pH 7.5 and temperature 37ºC, and the corresponding chemical shift plots as a function of time are shown in this figure. (**a** & **b)** Hɛ proton, (**c** & **d**) Hδ proton, (**e & f**) Hβ1 proton, (**g & h**) Hβ2 proton and (**i** & **j**) Hα proton. Monoexponential decay kinetic equation (equation 2 in the methods section) has been fitted to all the chemical shift plots.


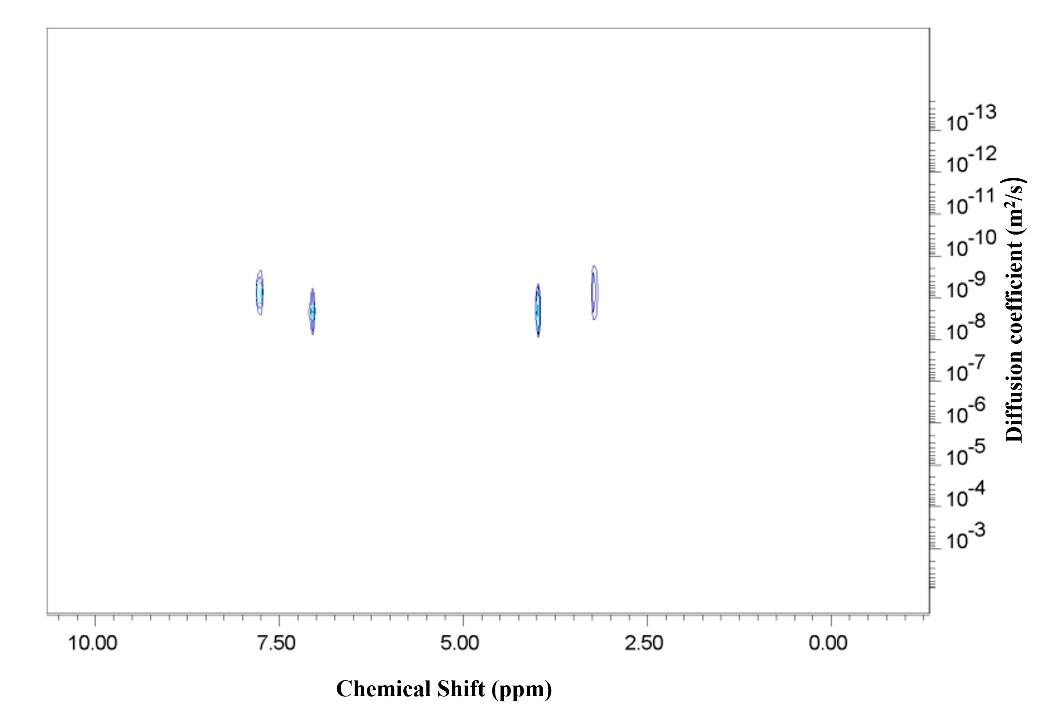


**Figure S6**: DOSY spectrum of 25 mM L-His in water at pH 7.5 and temperature 37ºC, after two hours of incubation.


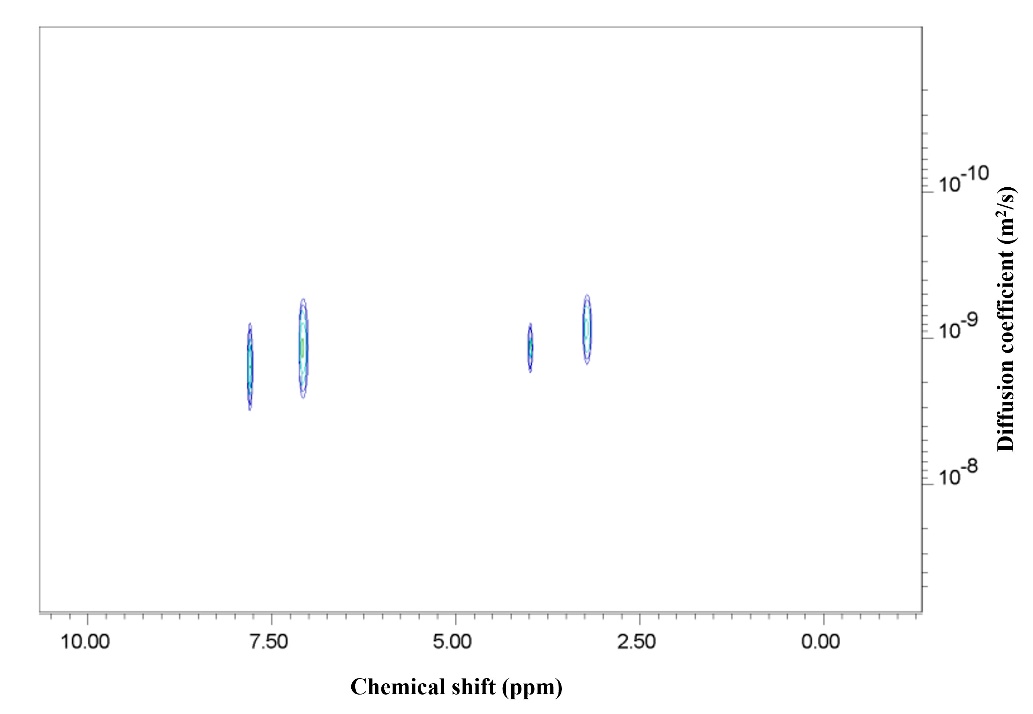


**Figure S7**: DOSY spectrum of 1 mM L-His in water at pH 7.5 and temperature 37ºC, after twenty-four hours of incubation.

**References**

1. Johanna Hjalte *et. al.* Aggregation behavior of structurally similar therapeutic peptides investigated by 1 H NMR and all-atom molecular dynamics simulations, Molecular Pharmaceutics, **19 (3)**, 904-917 (**2022)**
