# Supplementary figures and images for: The self-assembly of l-histidine might be the cause of histidinemia
Source: Sci Rep. 2023 Oct 14;13:17461. doi: 10.1038/s41598-023-44749-5 (PMC10576791; doi:10.1038/s41598-023-44749-5)

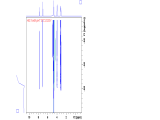

Supplement: Supplementary file 2 — Supplementary Information 2. [file 41598_2023_44749_MOESM2_ESM.zip › DOSY Data/10_1mM L-His DOSY/pdata/1/thumb.png]

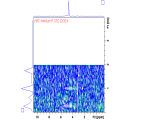

Supplement: Supplementary file 2 — Supplementary Information 2. [file 41598_2023_44749_MOESM2_ESM.zip › DOSY Data/10_1mM L-His DOSY/pdata/2/thumb.png]

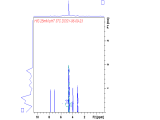

Supplement: Supplementary file 2 — Supplementary Information 2. [file 41598_2023_44749_MOESM2_ESM.zip › DOSY Data/7_25mM L-His DOSY/pdata/1/thumb.png]

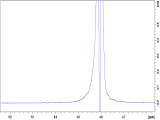

Supplement: Supplementary file 3 — Supplementary Information 3. [file 41598_2023_44749_MOESM3_ESM.zip › Raw NMR Data-L-His-Sunilkumar P N/NMR Data-25mM L-His at 20C pH 7-8/10/pdata/1/thumb.png]

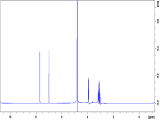

Supplement: Supplementary file 3 — Supplementary Information 3. [file 41598_2023_44749_MOESM3_ESM.zip › Raw NMR Data-L-His-Sunilkumar P N/NMR Data-25mM L-His at 20C pH 7-8/11/pdata/1/thumb.png]

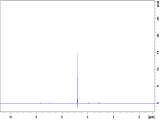

Supplement: Supplementary file 3 — Supplementary Information 3. [file 41598_2023_44749_MOESM3_ESM.zip › Raw NMR Data-L-His-Sunilkumar P N/NMR Data-25mM L-His at 20C pH 7-8/12/pdata/1/thumb.png]

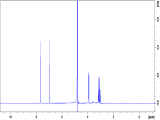

Supplement: Supplementary file 3 — Supplementary Information 3. [file 41598_2023_44749_MOESM3_ESM.zip › Raw NMR Data-L-His-Sunilkumar P N/NMR Data-25mM L-His at 20C pH 7-8/13/pdata/1/thumb.png]

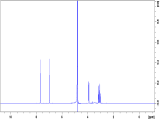

Supplement: Supplementary file 3 — Supplementary Information 3. [file 41598_2023_44749_MOESM3_ESM.zip › Raw NMR Data-L-His-Sunilkumar P N/NMR Data-25mM L-His at 20C pH 7-8/14/pdata/1/thumb.png]

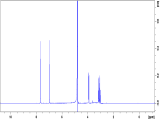

Supplement: Supplementary file 3 — Supplementary Information 3. [file 41598_2023_44749_MOESM3_ESM.zip › Raw NMR Data-L-His-Sunilkumar P N/NMR Data-25mM L-His at 20C pH 7-8/15/pdata/1/thumb.png]

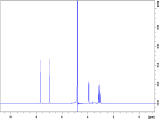

Supplement: Supplementary file 3 — Supplementary Information 3. [file 41598_2023_44749_MOESM3_ESM.zip › Raw NMR Data-L-His-Sunilkumar P N/NMR Data-25mM L-His at 20C pH 7-8/16/pdata/1/thumb.png]

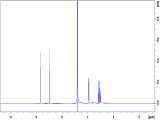

Supplement: Supplementary file 3 — Supplementary Information 3. [file 41598_2023_44749_MOESM3_ESM.zip › Raw NMR Data-L-His-Sunilkumar P N/NMR Data-25mM L-His at 20C pH 7-8/17/pdata/1/thumb.png]

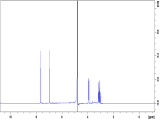

Supplement: Supplementary file 3 — Supplementary Information 3. [file 41598_2023_44749_MOESM3_ESM.zip › Raw NMR Data-L-His-Sunilkumar P N/NMR Data-25mM L-His at 20C pH 7-8/18/pdata/1/thumb.png]
